# Generation of a stable mouse endometrial tumor-derived cell line using conditional reprogramming

**DOI:** 10.1186/s40659-026-00706-w

**Published:** 2026-06-11

**Authors:** Tang Ansu Zhang, Rong Zhao, Yu Zhang, Hongbo Wang

**Affiliations:** 1 Department of Obstetrics and Gynecology, Union Hospital, Tongji Medical College, Hua Zhong University of Science and Technology, Wuhan, China; 2 Department of Gynecology, Xiangya Hospital, Central South University, Changsha, China

**Keywords:** EC, Genetically engineered mice, Conditional reprogramming, Cell line, Gene mutation

## Abstract

**Background:**

Endometrial cancer (EC) is a common malignancy of the female reproductive tract worldwide. While comprehensive genomic analyses have identified multiple genetic alterations in EC, disease models remain relatively rare, limiting the ability to conduct disease studies. Establishing a hybrid in vitro and in vivo model of EC and comparing it to its in vivo counterpart could greatly advance research into EC mechanisms and treatment strategies.

**Methods:**

We established genetically engineered EC mice (*Pten* ^*flox/+*^; *Stk11* ^*flox/flox*^; *LTF-Cre* (PSC)) and constructed a corresponding epithelial cell line from isolated uterine tumor tissues using conditional reprogramming techniques. The cell line was successfully passaged, frozen, and stored in a biobank, providing a valuable resource for future EC research.

**Results:**

The PSC-derived cell line stably retained the mutant alleles of *Pten* and *Stk11* during passaging, preserving the genetic profile of the original tumor. In addition, we performed various functional assays on isolated mouse cell linesand formed tumors after subcutaneous implantation of cells in immunodeficient mice and C57BL/6 mice with an intact immune system. Hematoxylin and eosin (H&E) staining and immunohistochemical staining were used to characterize the histopathology and biomarkers of the subcutaneously implanted tumors, which were highly similar to those of the parental mouse uterine tumors.

**Conclusions:**

The isolated mouse cell line provides a powerful platform for basic and translational EC studies, matching well with the corresponding in vivo models. The fidelity of the cell line to human-relevant genetic alterations and tumor biology makes it a valuable tool for investigating EC pathogenesis and evaluating novel therapeutic approaches.

**Supplementary Information:**

The online version contains supplementary material available at 10.1186/s40659-026-00706-w.

## Background

Endometrial cancer (EC) is the most common gynecological cancer and the sixth most common cancer among women worldwide. By 2020, approximately 417,000 new cases and more than 90,000 related deaths were reported globally [1]. Over the past few decades, the overall incidence has increased by 132% [2]. While hysterectomy cures most early-stage EC cases, the prognosis for advanced cases is poor [3]. Therefore, improving our understanding of the molecular mechanisms of EC is crucial for developing better treatments for patients with adjuvant refractory, advanced, and recurrent EC [4–6]. To achieve this increased understanding of EC, in vitro studies are needed.

There are established and well-known drivers that are present and highly prevalent in human patients with EC. STK11 was initially identified as a tumor suppressor gene that is mutated in Peutz–Jeghers syndrome (PJS), a hereditary syndrome closely associated with EC. Patients with PJS have a relatively high risk of developing epithelial malignancies, and women with PJS face a 9% increased lifetime risk of developing EC [7, 8]. Recent studies have shown that 21% of primary endometrial tumors also exhibit loss of STK11 protein expression. This is speculated to be an important reason for the occurrence and malignant transformation of EC [9, 10]. PTEN, an important tumor suppressor factor, is mutated in > 70% of ECs, highlighting its central importance in endometrial tumorigenesis [6]. Consistent with our expectations, the concomitant deletion of PTEN and STK11 in the mouse endometrial epithelium resulted in the classic pathological features of endometroid adenocarcinoma. However, the use of genetically engineered mouse models (GEMMs) is restricted by high cost and complex breeding processes [11].

Tumor cell lines are essential tools for studying human cancers both in vitro and in vivo. Recent EC research has relied heavily on established cell lines derived from human EC tumors [12–14]. These cell lines are widely used to study the biological behavior, pathogenesis, and drug sensitivity/resistance of EC. In vivo tumorigenesis experiments typically involve immunodeficient nude mice; however, these xenograft models are not well suited for studying the tumor microenvironment and antitumor immune response, limiting the study of immune-related therapies. Unfortunately, murine-EC cell lines for transplantation into immunocompetent mice are scarce.

Conditional reprogramming, which allows the indefinite proliferation of multiple epithelial cell types in vitro by coculturing with irradiated fibroblast feeder cells and the Rho kinase (ROCK) inhibitor Y-27,632, has been widely used in a variety of diseases [15]. With advances in genetic engineering, researchers have developed genetically engineered mouse models of EC [16–18]. Long-term expansion of EC epithelial cells from these models offers a new platform for studying EC physiology and its underlying mechanism. Here, we used a genetically engineered PSC mouse model to culture primary cells from mouse uterine tumors using conditional reprogramming (CR) technology to establish immortalized mouse EC epithelial cell lines that retain their tumorigenic and genetic characteristics. These cells provide valuable tools for functional studies, offering new insights into endometrial tumorigenesis and potential therapeutic approaches.

## Methods

### Genetically engineered mouse models

*Pten* ^*flox/flox*^ (P), *Stk11*^*flox/flox*^ (S), and *LTF-Cre* (C) mice were obtained from Shanghai Model Organisms (Shanghai, China). To generate PSC mice, we first crossed *Pten* ^*flox/flox*^ mice with *Stk11*^*flox/flox*^ mice to produce *Pten* ^*flox/flox*^; *Stk11*^*flox/flox*^ (PS) mouse offspring. These mice were then crossed with *LTF-Cre* mice to create PSC (*Pten* ^*flox/+*^; *Stk11*^*flox/flox*^; *LTF-Cre*) mice. All the mice were bred and maintained under standard conditions. To genotype each mouse, DNA samples were extracted from the tail using a One-Step Mouse Genotyping Kit (PD101-01, Vazyme, Nanjing, China). The genotypes and DNA recombination were analyzed by using genomic PCR (Table S1). Mice were monitored weekly for palpable tumors and euthanized via cervical dislocation when either the genetically modified mice reached 4 months of age or when the tumors grew larger than 1 cm^3^ (whichever occurred first). After being anesthetized with 1% pentobarbital sodium (P3761, Sigma‒Aldrich, Shanghai, China), the mice were sacrificed via cervical dislocation. The diseased uteri were then removed via necropsy, and viable endometrial epithelial cells were isolated. Some uterine tissues were fixed in formalin, while others were snap-frozen in liquid nitrogen and cryopreserved at − 80 °C.

The animal study protocol was approved by the Institutional Animal Care and Use Committee of Tongji Medical College, Huazhong University of Science and Technology (protocol code 2022-S019 and date of approval).

## Isolation and culture of primary endometrial epithelial cells

The obtained sterile tumor tissue was thoroughly washed with cold sterile 1X phosphate-buffered saline (PBS; G4202, Servicebio, Wuhan, China) to remove blood and then finely cut into the smallest possible fragments using ophthalmic scissors. These tissue fragments were incubated with 0.1% collagenase IV (40510ES60, YEASEN, Shanghai, China) at 37 °C for 15 min on a shaking platform. To stop the enzymatic reaction, an equal volume of complete medium was added. The tissue was then aspirated repeatedly using a 5 mL syringe to obtain a single-cell suspension. This suspension was filtered through a 70 μm cell sieve into a 50 mL centrifuge tube and centrifuged at 300 × g for 5 min at 4 °C, after which the supernatant was discarded. The resulting cell pellet was resuspended in Dulbecco’s modified Eagle’s medium/Nutrient Mixture F-12 1:1 medium (DMEM/F-12 1:1; L310KJ, BasalMedia; Shanghai, China) + 1% penicillin‒streptomycin (PS; E607011-0100, Sangon, Shanghai, China**) +** 10% fetal bovine serum (FBS; F103, Vazyme, Nanjing, China) and transferred to a T25 cell culture flask containing a feeder layer of cells cultured at 37 °C with 5% CO_2_ in a humidified incubator.

The tissue-derived endometrial epithelial cells were cultured using the CR cell method. This culture involved a standard combination of Swiss-3T3-J2 mouse fibroblasts as feeder cells, along with 10 µM ROCK inhibitor Y-27,632 (SC0326, Beyotime, Shanghai, China). The primary cell culture medium Complete F Medium was prepared by mixing DMEM/F-12 3:1 (BasalMedia, Shanghai, China) with 5 µg/mL insulin (11376497001, Sigma‒Aldrich, Shanghai, China), 250 ng/mL amphotericin B (A429759, Sangon, Shanghai, China), 10 µg/mL gentamicin (A462549, Sangon, Shanghai, China), 0.1 nM cholera toxin (9012-63-9, Yeasen, Shanghai, China), 0.125 ng/mL epidermal growth factor (Z03920-100, Genscript, Nanjing, China), 25 ng/mL hydrocortisone (IH1850, Solarbio, Beijing, China), 1% PS, and 10% FBS.

## Immunohistochemistry and pathological examination

We used 10% formalin (G1101, Servicebio, Wuhan, China) for the immunohistochemical staining of fixed, embedded uterine tumor tissues. The tissues were routinely processed and embedded, and 5-µm-thick sections were stained with H&E or subjected to immunohistochemistry (IHC). Photographs of the stained sections were captured using a light microscope (Zeiss, Oberkochen, Germany), and both H&E- and IHC-stained sections were independently evaluated by a professional pathologist in a blinded manner.

## Western blot analysis

Adherent cells were washed with cold sterile 1X PBS three times and extracted with RIPA lysis buffer (P0013B, Beyotime, Shanghai, China) supplemented with 2% cocktail (C0001, TargetMol, Boston, USA) and 0.2% PMSF (ST505, Beyotime, Shanghai, China). After being lysed on ice for 1 h, the total protein was centrifuged at 14,000 rpm for 15 min. The protein concentration was determined using a Bicinchoninic Acid (BCA) protein assay kit (P0012, Beyotime, Shanghai, China). Each sample was separated using sodium dodecylsulfate (SDS)-polyacrylamide gel electrophoresis on 10% gels (PG122, Yamei, Shanghai, China) and transferred to a polyvinylidene difluoride (PVDF) membrane (03010040001, Merck, Shanghai, China). The membranes were blocked in 5% nonfat milk (P0216, Beyotime, Shanghai, China) at room temperature for 1 h and then washed with 1% TBST (ST671, Beyotime, Shanghai, China) and incubated with primary antibody at 4 °C overnight. The next day, the membranes were washed with 1% TBST and incubated with HRP-conjugated AffiniPure goat anti-rabbit IgG (H + L) (1:10,000; SA00001-2, Proteintech, Wuhan, China) and HRP-conjugated AffiniPure goat anti-mouse IgG (H + L) (1:10,000; SA00001-1, Proteintech, Wuhan, China) secondary antibodies at room temperature for 1 h. Finally, images were captured with ChemiDoc MP software using an enhanced chemiluminescence (ECL) kit (PMK0448, BIOPRIMACY, Wuhan, China).

## Primary antibodies

The anti-STK11 (1:1,000 for WB and 1:200 for IHC, 3047T, D60C5) antibody was purchased from Cell Signaling Technology (Danvers, USA). Anti-ACTB (1:8,000 for WB, RP02968LQ), anti-GAPDH (1:10,000 for WB, A19056) and anti-PTEN (1:1,000 for WB, A21892) antibodies were purchased from ABclonal Technology (Wuhan, China). Anti-F4/80 (1:10,000 for IHC, 29414-1-AP), anti-FCN1 (1:200 for IHC, 11775-1-AP), anti-CD66B (1:1,000 for IHC, 30875-1-AP), anti-CXCR2 (1:200 for IHC, 19538-1-AP), anti-Vimentin (1:50 for IHC and 1:200 for IF, 10366-1-AP), anti-E-cadherin (1:50 for IHC and 1:200 for IF, 20874-1-AP), anti-pan-CK (1:200 for IF, 26411-1-AP), and anti-Snail (1:100 for IF, 13099-1-AP) antibodies were purchased from Proteintech Group (Wuhan, China). Anti-CD8 (1:100 for IHC, GB12068-100) and anti-CK19 (1:100 for IHC, GB11197) antibodies were purchased from Servicebio (Wuhan, China).

### Chromosome characterization

PSC01 cells in the logarithmic growth phase were treated with 10 µg/mL of colcemid (S204-010, BDBIO, Shanghai, China) for 4 h. The cells were harvested, and chromosomes from the nuclei of the cells were isolated with 0.075 mol/L KCl (P5405, Sigma‒Aldrich, Shanghai, China) and fixed in acetic acid/methanol (1:3, 10000208, 80080360, HUSHI, Shanghai, China). The cell suspension was placed on a glass slide and stained with Giemsa (G5637, Sigma‒Aldrich, Shanghai, China). The slides were observed under a microscope (Zeiss, Oberkochen, Germany), and microphotographs of the chromosomes were taken.

## Morphology of cancer cells

Cellular morphology was visually examined using a light microscope (Zeiss, Oberkochen, Germany). For a detailed cytological assessment, Giemsa staining was subsequently performed. Briefly, cells were seeded onto glass slides at a density of 1 × 10^5^ cells/mL and air-dried. The samples were fixed with 100% methanol or 10 min at room temperature, followed by staining with Giemsa reagent (G1079, Servicebio, Wuhan, China) at 37 °C for 15 min. After being rinsed and air-dried, the slides were dehydrated in xylene (10023428, HUSHI, Shanghai, China) for 5 min and permanently mounted with neutral resin. Representative images of both live observations and staining images are presented. The morphological assessment of cancer cells was conducted independently by two experienced pathologists who were blinded to the experimental groups. The evaluation was based on predefined criteria, including specific cell type identification, nuclear state, cellular size and shape, their respective proportions, and spatial distribution. Only cells that were jointly confirmed by both evaluators were considered for the final analysis.

## Flow cytometry

Feeder 3T3-J2 cells were removed by adding 0.25% trypsin (R001100, ThermoFisher, Shanghai, China) for 30 s, followed by washing with sterile 1X PBS. Afterward, 0.05% trypsin was added, and the samples were digested for 10–15 min. Epithelial cell suspensions were collected, washed twice with sterile 1X PBS, centrifuged to remove the supernatants, and resuspended in staining buffer (sterile 1X PBS with 1% FBS) at a concentration of approximately 1 × 10^6^ cells/mL. The cells were incubated with a PE-conjugated anti-mouse CD326 antibody (1:100; clone G8.8, Elabscience, Wuhan China) for approximately 30 min on ice. The cells were subsequently washed twice with staining buffer and analyzed using a Sony 7000 flow cytometer. CD326 expression was then assessed.

### Enzyme-linked immunosorbent assay (ELISA) evaluation

Whole blood samples were allowed to clot at room temperature for 2 h or at 4 °C overnight, followed by centrifugation at 1000 × g for 20 min. The supernatant (serum) was carefully collected for subsequent analysis. Circulating CA125 levels were quantified using a commercial mouse CA125 ELISA kit (ELK1479, ELK Biotechnology, Wuhan, China) according to the manufacturer’s instructions. Each experimental group (Control and GEMM) consisted of six mice.

### Cell line authentication service

Cell line DNA was extracted using the Axygen Genome Extraction Kit (Axygen Biosciences, Hangzhou, China) following the manufacturer’s instructions. The DNA was then amplified using a multiplex PCR system by following a four-color fluorescent probe qPCR amplification protocol for humans, mice, rats, and hamsters. The results were detected using an ABI7500 real-time fluorescence quantitative assay (ThermoFisher, Shanghai, China).

### Cellular immunohistochemistry

Round coverslips were prepared, and the cells were cultured for 3 days, after which they were washed with sterile 1X PBS, fixed with cold methanol for 8–10 min, washed again with sterile 1X PBS, and then fixed with 4% paraformaldehyde (10010061, HUSHI, Shanghai, China) for 15–20 min. The coverslips were allowed to air-dry. IHC was performed using anti-Vimentin, anti-E-cadherin, and anti-CK19 antibodies. The anti-Vimentin and anti-E-cadherin antibodies were diluted 1:50, and the anti-CK19 antibody was diluted 1:100.

### Proliferation assay

PSC-01 cells were kept in culture for up to 40 passages (~ 5 months) without any signs of senescence. Therefore, PSC-01 cells at cell passage 40 (P40) were considered a permanent cell line and were used for subsequent experiments. Proliferation assays were conducted at cell passage 20 (P20) and P40. Cells were seeded on 96-well plates at a density of 2000 cells/well on Day 1. The culture medium was changed every three days, and the cells were harvested on Day 7. The doubling time was determined using the equation: doubling time = duration × log (2)/log (final cell number) − log (initial cell number).

### Cell invasion and migration

PSC-01 cells were collected in serum-free culture medium to create a single-cell suspension. A total of 1 × 10^5^ cells were added to the upper layer of the Transwell chambers, while 600 µL of DMEM/F-12 culture medium containing 20% FBS was added to the lower layer of the chambers. The cells were routinely cultured for 6, 12, or 24 h, fixed with 4% paraformaldehyde, stained with 0.3% crystal violet (Y268091, Beyotime, Shanghai, China), photographed, and counted under a microscope.

### Colony formation assay

To perform the colony formation assays, 500–2000 cells per well were seeded in 6-well plates and cultured at 37 °C for 14 days. At the end of the experiment, colonies (with at least 50 cells/colony) were stained with 0.3% crystal violet and directly counted on the plate. Each measurement was performed in triplicate, and each experiment was conducted at least three times.

### β-galactosidase staining

To evaluate senescence, high-passage PSC-01 cells were plated at 5 × 10^5^ cells per well in 6-well plates and grown to confluence. The cells were fixed in staining fixative, washed once with sterile 1X PBS, and stained for β-galactosidase (C0602, Beyotime Shanghai, China).

### Cytotoxicity assay

The cytotoxic effects of the chemotherapeutic drugs carboplatin, cisplatin, paclitaxel, and azithromycin on the PSC cell line were compared. Carboplatin, cisplatin, paclitaxel, and azithromycin were all purchased from MedChemExpress (MCE) (HY-17393, HY-17394, HY-B0015, HY-17506, Shanghai, China). For the cytotoxicity assay, 5,000 cells suspended in complete medium were seeded into each well of a 96-well plate. The next day, the cells were treated with different concentrations of carboplatin (5–2500 µM), cisplatin (5–800 µM), paclitaxel (1–1000 nM), and azithromycin (5–100 µM). After the cells were incubated with different chemotherapeutic drugs for 24–48 h, medium supplemented with 10% Cell Counting Kit-8 (CCK-8) reagent (C0005, TOPSCIENCE-tsbiochem, Shanghai China) was added. After 1 h of incubation, the percentage of cytotoxicity was calculated by measuring the absorbance value at 450 nm. The half maximal inhibitory concentration (IC50) of each chemotherapeutic drug for each cell line was determined by interpolating the values from the graph (% cytotoxicity vs. drug concentration).

### Tumorigenicity studies

BALB/c nude mice or C57BL/6 mice (4–6 weeks old) were purchased from CHARLES RIVER (Beijing, China) and inoculated with 5 × 10^6^ PSC-01cells, which were injected subcutaneously into the right back. The mice were housed in a pathogen-free IVC room at a temperature of 22 °C and a relative humidity of 30–70%. Tumor development was monitored using calipers every two days, and tumor size was assessed by length and width. The tumor volume was calculated using the formula V = length × (width^2^/2). After 6 weeks, when the tumor volume reached 1 cm³, the mice were euthanized using pentobarbital sodium followed by cervical dislocation. The tumors were then excised, and each allograft was processed for histological examination. The tumor sections were fixed in 10% formalin and embedded in paraffin. H&E staining was used to visualize tumor morphology, and IHC was performed to assess the expression of STK11. This study was approved by the Animal Use Protocol Committee of Tongji Medical College, Huazhong University of Science and Technology.

### Whole-exome sequencing

Genomic DNA was sent to OEbiotech (Shanghai, China) for DNA library construction. Sequencing was performed using an Illumina HiSeq 4000 platform, clean reads were aligned to the reference genome using BWA, and the results were converted to SAMtools format. Duplicates were removed using Picard, and data analysis was conducted using Qualimap software (reference genome version: GRCm38.p6). SNP and InDel analyses were performed using the Haplotype Caller module of GATK4 software on the basis of the alignment with the reference genome.

### Statistical analysis

The data are presented as the mean ± standard error of the mean (SEM). Differences between mean values were analyzed using Student’s t test, with *P* < 0.05 considered to indicate statistical significance. All the statistical calculations were performed with GraphPad Prism software.

## Results

### In vitro establishment of the PSC cell line

**Fig. 1:**
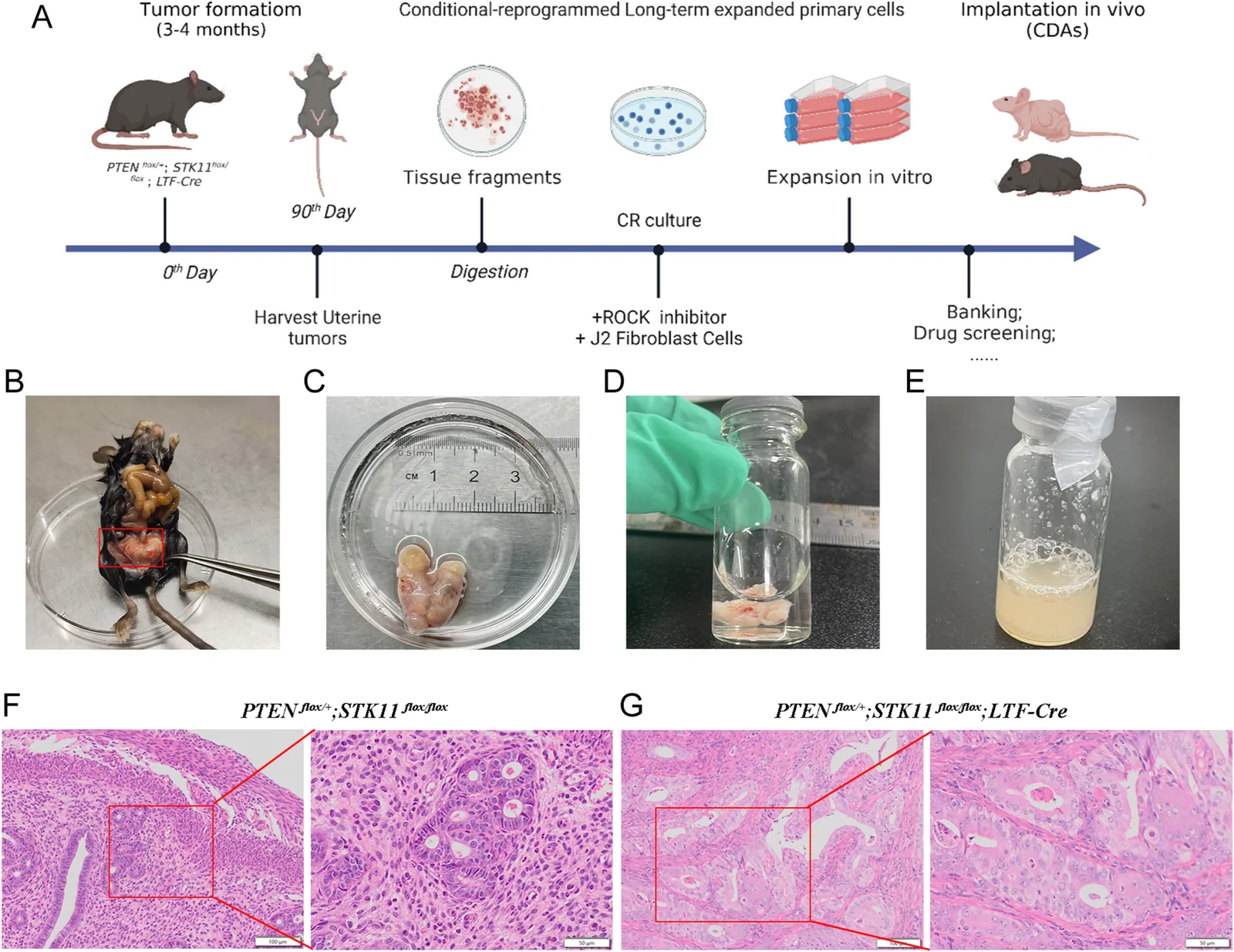
1 In vitro establishment of PSC cell line.** A** Experimental design for the establishment of PSC cell line.** B** Anatomical view of uterine tumors in 12-week-old Pten^flox/+^; Stk11^flox/flox^; LTF-Cre (PSC) mice.** C** Isolated uterine tumor.** D**,** E** Tissue digestion and separation.** F**,** G** Representative photomicrographs of H&E staining in tumor tissues. Scale bars = 200 μm

Cell lines established from primary tumors have been proven invaluable for elucidating the molecular events that lead to cellular transformation and for studying disease progression. While several genetically engineered mouse models have been developed for endothelial cancer, few reports exist on mouse EC cell lines. Therefore, we aimed to establish mouse EC cell lines from genetically engineered murine primary tumor tissues of spontaneous endometrial carcinoma (Fig. 1A). A mouse PSC cell line was successfully generated from spontaneous endometrial tumors of *Pten* ^*flox/+*^; *Stk11* ^*flox/flox*^; *LTF-Cre* mice at 17 weeks of age (Fig. 1B and E). Histological analysis of primitive uterine tumors derived from the cell line compared with the uteri of age-matched control mice is shown in Fig. 1F and G. The endometrial layer lost the well-differentiated glandular structure typical of a normal uterus and displayed irregular glands and moderately to poorly differentiated endometrial epithelial cells. In addition to being used for histopathological analyses, serum was collected. Quantitative ELISA revealed that circulating CA125 levels were significantly greater in the treated mice than in the control mice (Fig. S1A), corroborating the presence of an active tumor burden. These findings suggest that the uterine tumor was an invasive endometrial adenocarcinoma.

**Fig. 2:**
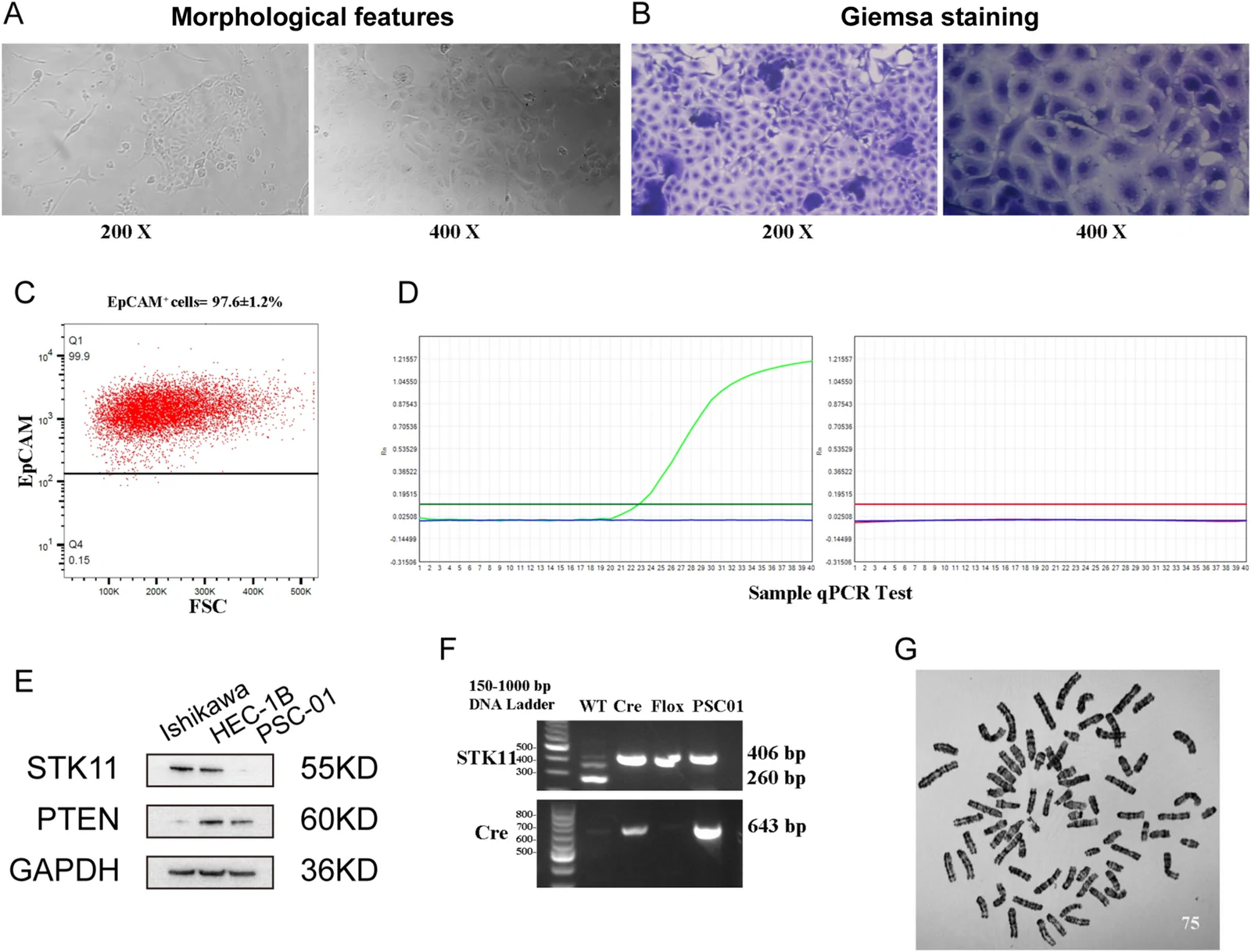
Cell physiology of PSC-01 cells.** A** Microphotographs (200×, 400×) of the cultured cells, showing typical cobblestone epithelial morphology.** B** Crystalline violet staining was performed to observe PSC-01 cell morphology.** C** Representative flow cytometry plot of EpCAM expression in PSC-01 cells. Data represent PSC-01 cells from more than three different generations.** D** qPCR resultsof the cell line, lane 1: mouse: HEX green, human: FAM-1 blue; lane 2: hamster: FAM-2 blue, rat: CY5 red.** E** Western blot analysis of PTEN and STK11 proteins in PSC-01 cells.** F** Genomic DNA isolated from PSC-01 cells derived from WT 、Flox(Pten flox/+; Stk11 flox/flox) or EC (Pten^flox/+^; Stk11^flox/flox^; LTF-Cre) mouse models. PSC-01 from the PSC mouse model contains the mutant STK11 gene (n = 3 individual animals in each genetic mouse model).** G** Identification and characterization of the chromosome content of the cell line were performed using the standard G-banding method. Representative karyotypes of PSC-01, at passage 40 are shown

The PSC cell line was isolated and cultured under reprogramming conditions on an irradiated feeder layer of JC-2 mouse fibroblasts in DMEM/F-12 medium supplemented with the ROCK inhibitor Y-27,632 to support long-term cell growth. When endothelial epithelial cells isolated from mouse uterine tumor tissue samples were inoculated onto feeder layer cells, multiple colonies formed within 2–3 days. After transitioning to normal medium (DMEM/F-12 medium supplemented with 10% FBS and 1% PS), the PSC cell line was continuously passaged more than 40 times without any signs of senescence (Fig. S1B). Microscopic examination revealed that adherent PSC-01 cells exhibited a classic cobblestone epithelial morphology at low magnification (200×), with individual cells possessing clearly defined round nuclei observable under higher magnification (400×) (Fig. 2A). The culture displayed a heterogeneous cellular composition, wherein cobblestone-like epithelial cells were primarily concentrated in the central region, while spindle-shaped, fibroblast-like cells were predominantly localized at the periphery. Cytological evaluation by Giemsa staining further revealed pronounced nuclear atypia, including nuclear enlargement, hyperchromasia, uneven chromatin distribution, and an abnormally high nucleus-to-cytoplasm (N/C) ratio, in PSC-01 cells (Fig. 2B). These morphological features are consistent with established cytopathological hallmarks of malignant transformation and support the neoplastic nature of the established cell line. The epithelial nature of the PSC-01 cells was confirmed through flow cytometry, which revealed that approximately 99% of the cells expressed the epithelial-specific surface marker EpCAM (Fig. 2C). Cellular DNA was extracted and amplified by a multiplex qPCR system with species-specific fluorescent probes (human, mouse, rat, and hamster). Detection was performed on an ABI 7500 Real-Time PCR System (ThermoFisher, USA). The amplification results shown in Fig. 2D confirmed the presence of only mouse cell DNA, with no contamination from a rat, hamster or human source, indicating that the cell line originated from a mouse. We designated this cell line PSC-01 and deposited it in the China Typical Culture Biological Conservation Center for long-term storage.

### Biological identification of the PSC-01 cell line

We examined the genotypes of the cell line to determine their concordance with the origin of the uterine tumor. As shown in Fig. 2E F, PSC-01 cells retained a genotype consistent with that of the mouse of origin and lost the wild-type STK11 allele while retaining the wild-type PTEN allele. Consistent with these genotypic findings, PSC-01 cells did not express the STK11 protein; however, the heterozygous mutations in PTEN did not affect PTEN protein expression.

We also analyzed the karyotype of the mouse PSC cell line using standard cytogenetic G-banding to assess the chromosomal patterns in the tumor cells. As shown in Fig. 2G and Fig. S2A and B, the chromosome counts of the PSC-01 cell line ranged from 61 to 80. The degree of intracellular diversity varied widely.

### Genomic characterization of the PSC cell line and its original primary tumor

**Fig. 3:**
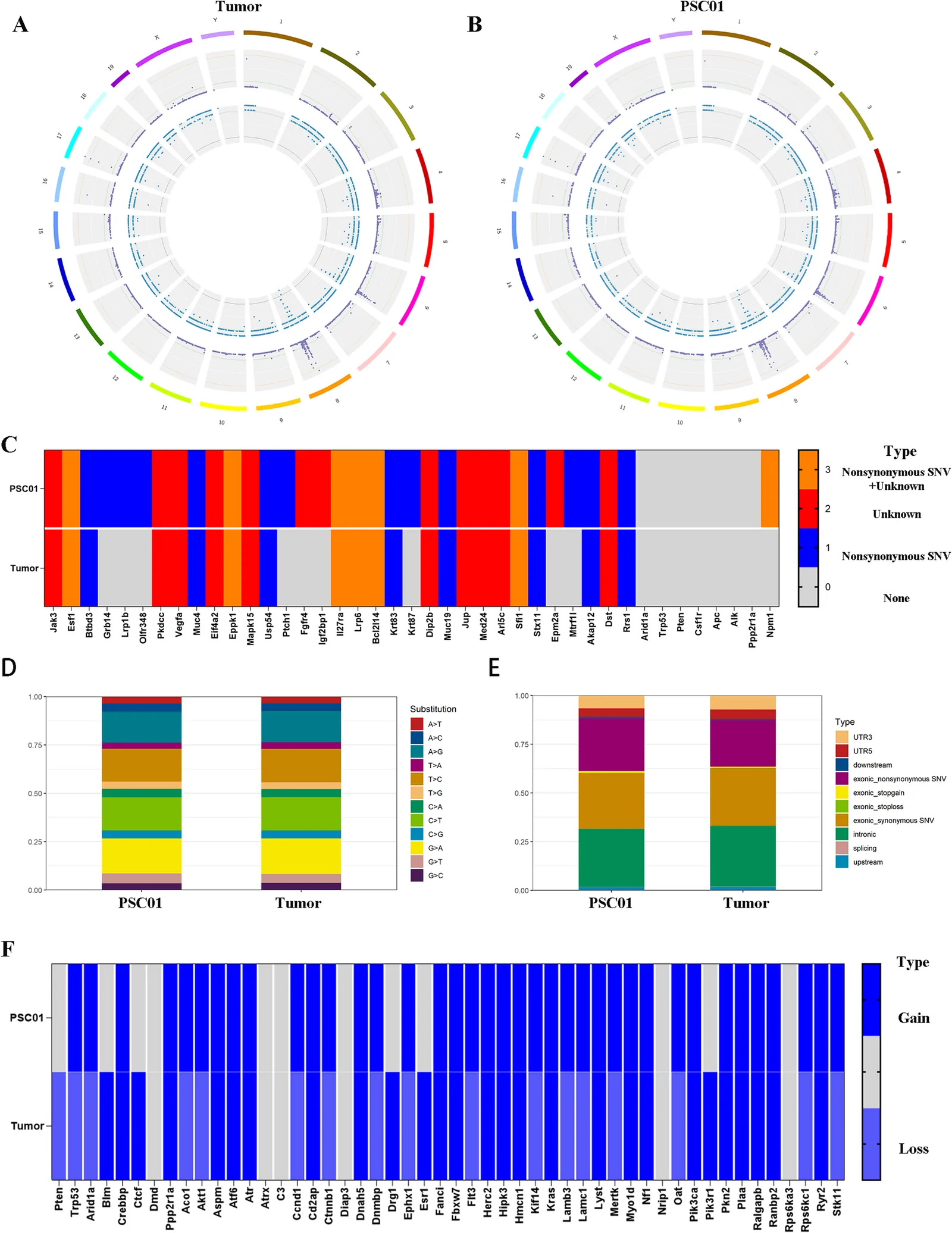
Genomic characterizations of PSC cell line and their original primary tumor.** A**,** B** Circos plots showing full mutation information in the primary tumor and in the corresponding PSC-01 cell line. Ring 1: chromosome; ring 2: purple loci indicate the distribution of genomic somatic variant density; ring 3: somatic CNV, red: copy number gain, blue: copy number deletion and green: normal copy number; ring 4: somatic SNV, CTX (brown), ITX (blue), INS (orange), DEL (dark red), DUP (light purple) and INV (blue).** C** Heatmap showing variations in gene mutations for the most frequently mutated genes. SNV: single-nucleotide variant.** D**,** E** Proportion of base substitutions and types of somatic mutations.** F** CNVs heatmap of EC driver genes in the primary tumor and the PSC-01 cell line

To further explore whether PSC-01 accurately represents the genetics and heterogeneity of primary malignancies, we performed a series of genomic comparisons between PSC-01 and the primary tumor samples from which they were derived. We obtained 80,483,454 and 72,983,252 paired raw reads (12.07 and 10.95 Gb, respectively), with average GC contents of 47.06% and 47.00%, respectively. The Q20 and Q30 values for the two groups were 97.83% and 93.60% and 97.59% and 93.10%, respectively, indicating high-quality sequencing data. The full mutation information detected in the Circos plot was integrated. As shown in Fig. 3A and B, the PSC-01 cells largely recapitulated the primary tumor. In addition, mutation profiling revealed that the PSC01 cell line retained most of the mutations observed in primary mouse EC tumors, including mutations in *Jak3*,* Sfi1*,* Stx11*, and *Muc19* (Fig. 3C and Table S2). PSC-01 cells and primary tumors also displayed similar overall trends in terms of point mutation type, SNP distribution, and InDels (Fig. 3D and E). Moreover, a search of the DriverDBv4 database revealed that the CNVs of common driver genes in EC, such as *FBXW7*,* PIK3CA*,* NF1*, and *KRAS* (human–mouse species homologous gene conversion), were highly similar between PSC-01 and the primary tumor (Fig. 3F). Overall, whole-exome sequencing (WES) analysis revealed that the genomic mutational mapping features of the established PSC-01 cells were highly similar to those of their matched primary tumors. These findings suggest that PSC-01 cells can partially represent the genetics of primary malignant tumors and hold potential value in translational applications.

### Biological properties of the PSC cell line

**Fig. 4:**
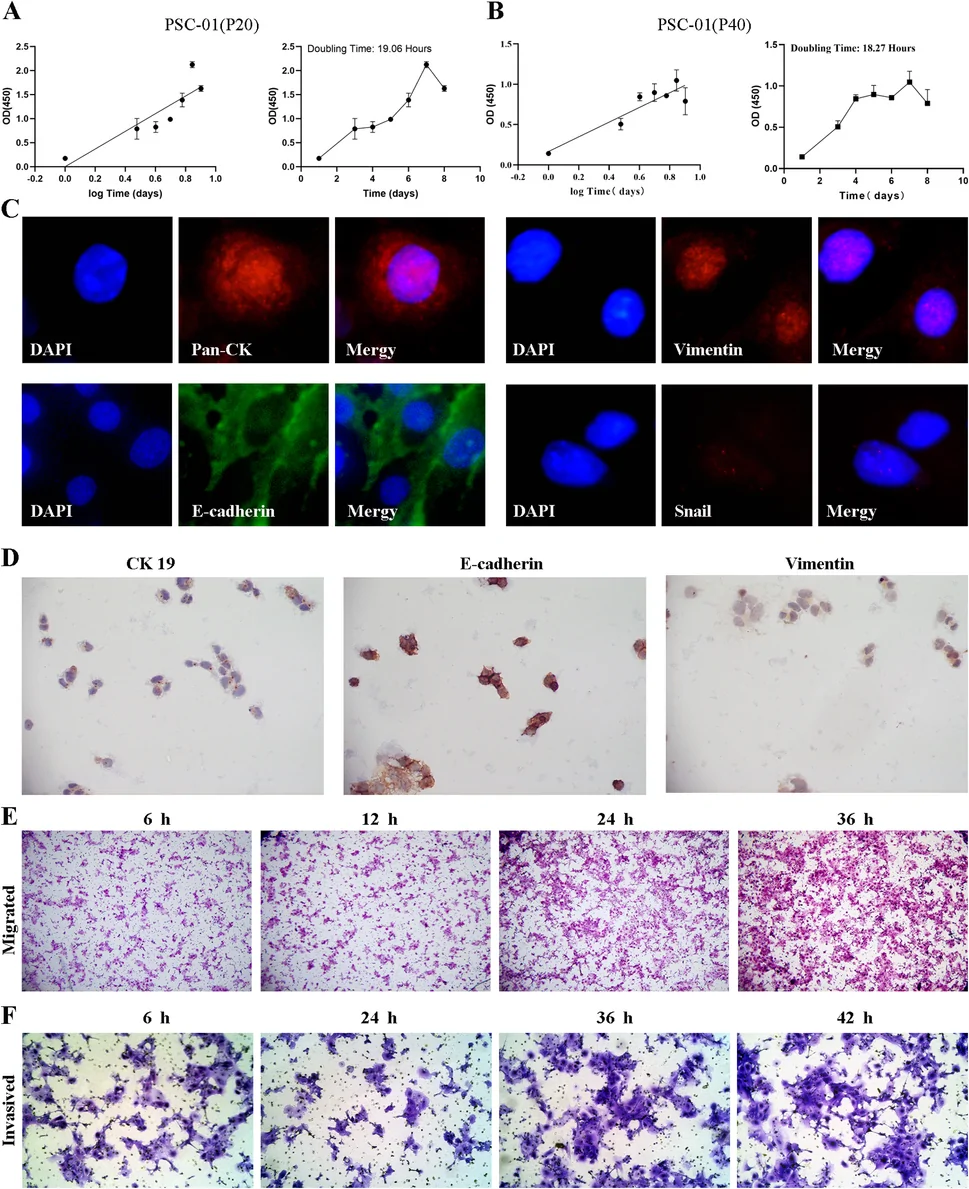
Biological properties of the mouse PSC-01 cell line.** A**,** B** Cells were seeded on 96-well plates and their growth was monitored daily by measuring absorbance after incubation with the CCK8 reagent. Data are presented as the mean absorbance of six replicates ± standard error. The doubling time = duration × log (2)/log (final cell number) − log (initial cell number).** C** Protein expression of pan-CK, Vimentin|, E-cadherin, and Snail on mouse PSC-01 cell lines was assessed by fluorescence microscopy. Scale bar: 50 µm** D** Micrographs of immunohistochemical staining of CK19, E-cadherin, and Vimentin in mouse PSC-01 cells (100×).** E** Migration potential was measured using a Transwell cell culture chamber (40×).** F** Invasion potential was measured using a Transwell cell culture chamber with Matrigel added (100×)

The growth kinetics of the PSC-01 cell line at P20/P40 were examined using a CCK-8 assay, which revealed that the proliferation rate of PSC-01 cells decreased and that the proliferation rate remained stable across different generations of PSCs (Fig. 4A and B). Using immunofluorescence microscopy, we observed the presence of the tumor marker pan-CK, the epithelial marker E-cadherin, and the mesenchymal markers Vimentin and Snail in the PSC-01 cell line. The fluorogram results suggested that the PSC-01 cells were tumor epithelial cells (Fig. 4C and D). We further assessed the invasive and migratory abilities of PSC-01 cells using a Transwell assay. Cells that migrated to the basal side of the membrane were observed and photographed at 100 × magnification at different time points (Fig. 4E F).

### Tumorigenicity assay of the PSC cell line

**Fig. 5:**
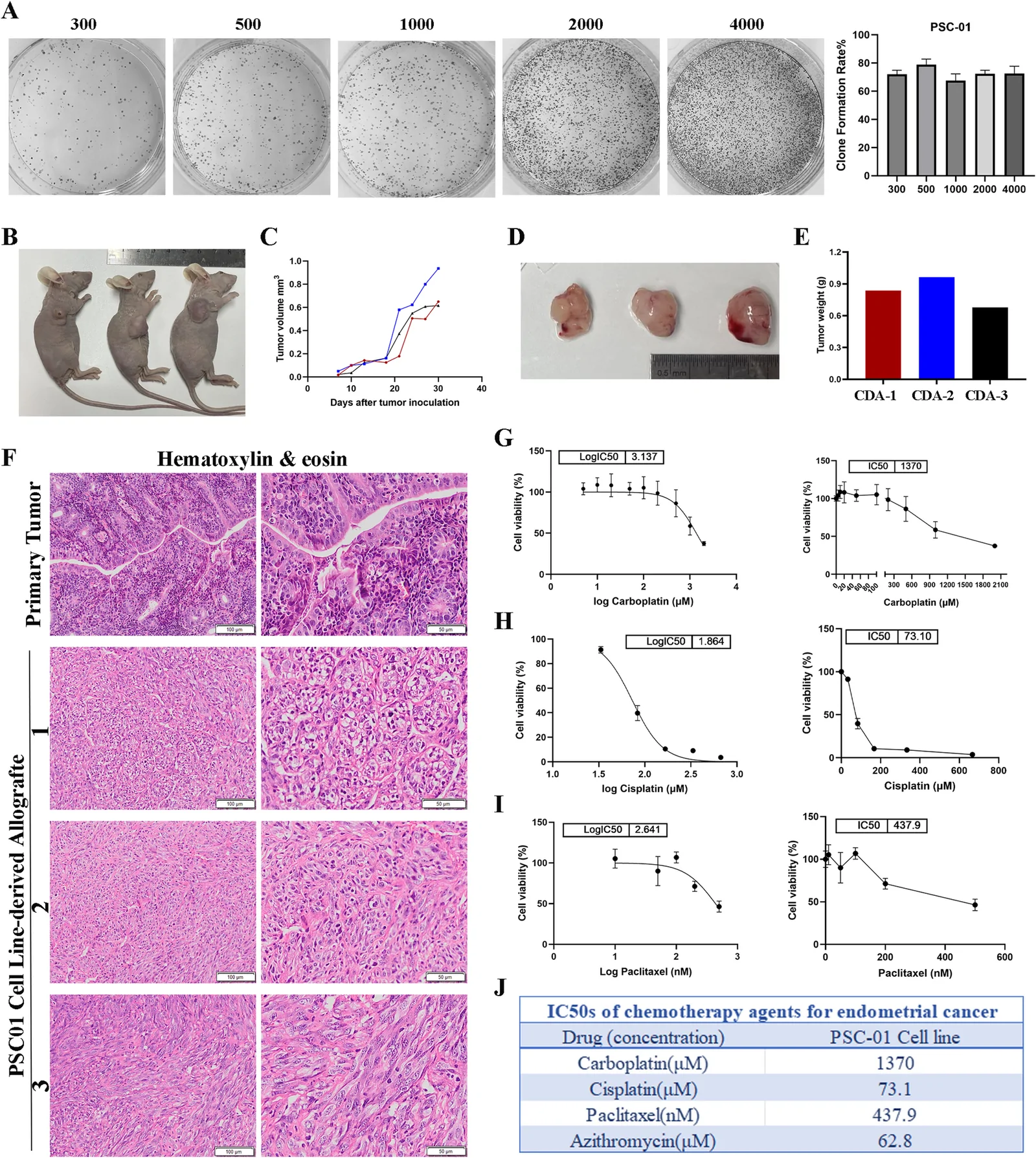
Tumorigenic properties of PSC-01 cells.** A** Colony-formation assay was used to assess the oncogenic potential of PSC-01 cells.** B** A tumorigenicity assay was performed on nude mice to evaluate the tumorigenic capacity of PSC-01 cells.** C** Growth curves of subcutaneous graft tumors measured three times a week.** D** Gross appearance of allograft tumors formed in BALB/C-nude mice harboring PSC-01 cells.** E** Weight of allograft tumors was measured.** F** H&E stained sections of tumors derived from PSC-01 cells after subcutaneous implantation, compared to primary uterine tumors. Scale bars: 100 µm; 50 µm.** G**,** H**,** I** Folding graphs showing the cell viability of PSC-01 cells treated with Carboplatin, cisplatin and paclitaxel.** J** IC50 values of the tested chemotherapeutic agents. Data are presented as the mean ± SD from three independent experiments for each drug

To further characterize the tumorigenic ability of PSC-01, we conducted colony formation and tumorigenicity assays in nude mice. We inoculated cell culture plates with varying numbers of PSC-01 cells and calculated the clonogenic rate, as shown in Fig. 5A. The number of colonies formed increased gradually with increasing number of inoculated cells; however, no significant difference in the clonogenic rate was detected. Furthermore, we tested the tumor initiation potential of PSC-01 cells via subcutaneous transplantation into the right scapulae of BALB/C-nude mice and C57BL/6 mice (Table S3, Fig. S3A and C). The fewer cells that were transplanted, the longer the latency period and the smaller the final tumor size (Fig. S3B and D). After the appropriate number of transplanted cells was selected, further subcutaneous injection of tumor cells into nude mice revealed that the PSC-01 cell line rapidly formed tumors within the skin of the mice. These tumors developed within two weeks, and the animals were sacrificed within one month after inoculation (Fig. 5B and E, Fig. S4A). Histopathological analyses of the tumors formed by the PSC-01 cell line and primitive uterine tumors are shown in Fig. 5F and Fig. S4B, and H&E staining revealed abundant cytoplasm, round or oval nuclei, prominent nucleoli, and significant heterogeneity, indicating histological similarity with poorly differentiated adenocarcinoma. Both the primary tumor and the cell line tumors histologically resembled high-grade EC.

### The PSC-01 cell line demonstrates tumorigenicity in the context of an intact immune system

The immunocompetent mouse tumor allograft model is a convenient tool to investigate the regulation of antitumor immunity in the tumor microenvironment (TME) in animals. With this in mind, we chose to examine the expression of several key signature genes of several immune cell types in subcutaneous tumors: macrophages (F4/80, FCN1), neutrophils (CD66B, CXCR2) and CD8. The results revealed that immune cell infiltration in subcutaneous tumors was not significant (Fig. S4C). However, the expression of CD8 in our GEMM primary tumor was significantly greater than that in the normal mouse uterus. As expected, STK11 was not detected in the cancer cells of the tumor tissue or subcutaneous tumors. (Fig. S4D).

### Evaluation of the efficacy of anticancer drugs against the PSC-01 cell line

The in vitro drug sensitivity of the PSC-01 cells to commonly used chemotherapy agents for EC treatment was tested. Using a CCK-8 assay, we evaluated the effects of carboplatin, cisplatin, paclitaxel, and azithromycin on PSC-01 cells. For carboplatin, the IC50 was 1370 µM, and the values ranged from 1162 to 1653 µM after 24 h of treatment. The IC50 was 73.1 µM after 24 h of treatment with cisplatin. For paclitaxel, the IC50 values ranged from 344.2 to 614.7 nM after 48 h of treatment. For azithromycin, the IC50 value was 62.8 µM after 24 h of treatment (Fig. 5G–J).

## Discussion

Genetically engineered mice have become among the most promising tools for studying the pathogenesis and progression of EC through the tissue-specific or spatially specific knockout of disease-associated oncogenes or overexpression of pro-oncogenes. These models have been increasingly used for pharmacokinetic testing of novel therapeutic agents [19–21]. However, owing to the high costs and time investment associated with these models, simpler and faster ways to establish biological models complement or assist in ongoing disease research are needed [22–25]. A faithful cell line that accurately represents a tumor is essential for understanding cancer biology and developing potential therapeutic strategies [26–28]. The establishment of tumor cell lines from such mouse models can serve as a valuable tool for basic and preclinical research, complementing genetically engineered mice [29].

The *STK11* tumor suppressor was identified in 1998 as a gene mutated in Peutz–Jeghers syndrome (PJS), an inherited cancer predisposition characterized by a high incidence of gastrointestinal polyposis and cancer, particularly at various anatomical sites, including the gastrointestinal tract, lung, and female genital tract. Women with PJS have a high incidence of cancer in the uterus (endometrium) and cervix [30, 31]. Loss of *STK11* has been observed in approximately 20% of primary ECs. Additionally, loss or mutation of *PTEN* (another tumor suppressor) occurs in more than 80% of endometrial adenocarcinomas [32, 33]. In mouse models of endometrial adenocarcinoma, loss of *STK11*, combined with heterozygous mutations in *PTEN*, is associated with marked invasion and rapid disease progression and spread, leading to early death [34]. In a PSC mouse model based on our previous construct, we observed a distinct tumorigenic phenotype in the endometrium, resembling aggressive endometrioid endometrial adenocarcinoma, where endometrial epithelial cells are typically moderately to poorly differentiated.

We used WES to obtain the genomic mutation profiles of the PSC-01 cell line and matched primary tumor tissues. Tail DNA from the same mice was used as a reference to identify the total number of SNPs and InDels. We found that the characteristics of the established PSC-01 cell line closely mirrored those of its corresponding primary tumor. On the one hand, the protein and DNA differences in PSC-01 cells were similar to those in the primary tumor; on the other hand, the gene mutations associated with EC were highly similar between PSC-01 cells and the primary tumors. However, we did not detect *Stk11* mutational alterations in PSC-01 cells, although *Stk11* protein levels were deficient. We propose the use of further combined transcriptomics sequencing analysis to explore this topic in future studies.

In this study, we established the PSC-01 cell line from PSC mouse uterine tumor tissues using CR technology [35, 36]. This cell line exhibited a typical epithelial cobblestone morphology with high expression of the tumor epithelial markers CK and E-cadherin, as confirmed by histochemical and immunofluorescence staining. PSC-01 cells not only expanded stably in vitro over a long period but were also passaged more than 70 times without signs of senescence, maintaining a genotype and growth characteristics similar to those of the tumor of origin. Currently, GEMMs are considered the most representative in vivo models of EC, with irreplaceable advantages for studying the occurrence, development, metastasis, and treatment of this disease [37–40]. However, their high cost and complexity limit their large-scale application [41, 42]. The established PSC-01 cell line can serve as a complementary EC GEMM. The advantages of PSC-01 cells over genetically engineered PSC mice (*Pten* ^*flox/+*^; *Stk11* ^*flox/flox*^; *LTF-Cre*) are as follows: lower cost, rapid tumor formation, consistent results, and real-time detection of dynamic tumor growth. PSC-01 cells can give rise to tumors in both immunodeficient and immunogenic nude mice, providing an excellent platform for preclinical studies to investigate the immune-related tumor microenvironment and the antitumor immune response to drugs, with significant translational implications.

## Conclusions

In summary, we successfully established a novel tumorigenic mouse EC cell line derived from a genetically engineered mouse model using CR technology. To the best of our knowledge, this is the first study of an immortalized mouse EC cell line generated via this approach. The mouse cells isolated in our study, along with the corresponding in vivo model, will serve as a valuable tool for both basic and preclinical research. This information will enhance our understanding of EC pathogenesis and facilitate the investigation of new therapies for this disease. These findings could provide further insights into the underlying mechanisms of endometrial tumorigenesis.

## Supplementary Information

Below is the link to the electronic supplementary material.

Supplementary Material 1.

Supplementary Material 2.

Supplementary Material 3.

Supplementary Material 4.

Supplementary Material 5

Supplementary Material 6.

## Data Availability

The datasets and materials used during the current study are available from the corresponding author on reasonable request.

## Abbreviations

EC: Endometrial cancer
CR: Conditional reprogramming
PSC: *Pten* ^*flox/flox*^ (P) mice, *Stk11*^*flox/flox*^ (S) mice, and *LTF-Cre* (C)
GEMM: Genetically engineered mouse model
WES: Whole-exome sequencing
PJS: Peutz–Jeghers syndrome
