# Supplementary material for: Generation of a stable mouse endometrial tumor-derived cell line using conditional reprogramming
Source: Biol Res. 2026 Jun 11;59:47. doi: 10.1186/s40659-026-00706-w (PMC13492030; doi:10.1186/s40659-026-00706-w)
Supplement: Supplementary file 1 [file 40659_2026_706_MOESM6_ESM.docx]

## Supplement Figure Legends

## Fig. S1 Analysis of CA125 in vivo and senescence marker in vitro. A Serum CA125 levels of control and GEMM mice. B Representative image of the β-galactosidase activity assessment of high-passage PSC-01 cells, no typical blue staining cells were found. Scale bars =100 μm.

## Fig. S2 Karyotype analysis of PSC-01 cells. A The chromosome spreads of PSC-01 cells. B Chromosome counting from metaphase PSC-01 cells.

**Fig. S3 Tumorigenic property of PSC-01 cells.** **A** Representative subcutaneous transplantation of tumors in BALB/C-nude mice. **B** Representative gross appearance of allograft tumors formed in BALB/C-nude mice carrying different orders of magnitude of PSC-01 cells. **C** Representative macroscopic of subcutaneous transplant tumor of C57BL/6 mice. **D** Representative gross appearance of allograft tumors formed in C57BL/6 mice carrying different orders of magnitude of PSC-01 cells.

**Fig. S4 Histological characteristics of subcutaneous tumors in C57BL/6 mice .A** A tumorigenicity assay was performed in C57BL/6 mice. **B** Representative H&E of tumors derived from PSC-01 cells implanted subcutaneously in C57BL/6 mice compared to primary tumors. Scale bars: 100 µm; 50 µm. **C** Histological analysis of macrophages (F4/80, FCN1) and neutrophils (CD66B, CXCR2) infiltration in subcutaneous tumor tissue. **D** Histological analysis of the expression of STK11 and CD8 in normal mouse uterus, primary tumor tissue, and subcutaneous tumors. As expected, STK11 was not detected in the cancer cells of the tumor tissue and subcutaneous tumors, Scale bars: 100 µm; 200 µm.
